# Psychological distress and personality factors in takotsubo cardiomyopathy

**DOI:** 10.1007/s12471-016-0861-3

**Published:** 2016-07-11

**Authors:** L. Smeijers, B.M. Szabó, W.J. Kop

**Affiliations:** 1 Department of Medical and Clinical Psychology, Center of Research on Psychology in Somatic diseases (CoRPS), Tilburg University, Tilburg, The Netherlands; 2 Department of Cardiology, Elisabeth-TweeSteden Hospital, Tilburg, The Netherlands

**Keywords:** Tako-Tsubo cardiomyopathy, Depression, Anxiety, Personality, Psychological distress, Apical ballooning syndrome

## Abstract

**Background:**

Takotsubo cardiomyopathy (TCC) is a transient condition characterised by severe left ventricular dysfunction combined with symptoms and signs mimicking myocardial infarction. Emotional triggers are common, but little is known about the psychological background characteristics of TCC. This study examined whether patients with TTC have higher levels of psychological distress (depressive symptoms, perceived stress, general anxiety), illness-related anxiety and distinct personality factors compared with healthy controls and patients with heart failure.

**Methods and Results:**

Patients with TCC (*N* = 18; mean age 68.3 ± 11.7 years, 77.8 % women) and two comparison groups (healthy controls: *N* = 19, age 60.0 ± 7.6, 68.4 % women and patients with chronic heart failure: *N* = 19, age 68.8 ± 10.1, 68.4 % women) completed standardised questionnaires to measure depression (PHQ‑9), perceived stress (PSS-10), general anxiety (GAD-7), illness-related anxiety (WI-7) and personality factors (NEO-FFI and DS-14). Psychological measures were obtained at 23 ± 18 months following the acute TTC event. Results showed that patients with TCC had higher levels of depressive symptoms (5.2 ± 5.2 vs. 2.5 ± 2.4, *p* = 0.039) and illness-related anxiety (2.1 ± 1.7 vs. 0.7 ± 1.3, *p* = 0.005) compared with healthy controls. Patients with TCC did not display significantly elevated perceived stress (*p* = 0.072) or general anxiety (*p* = 0.170). Regarding personality factors, levels of openness were lower in TCC compared with healthy controls (34.2 ± 4.3 vs. 38.2 ± 5.6, *p* = 0.021). No differences between TCC and heart failure patients were found regarding the psychological measures.

**Conclusions:**

TCC is associated with higher levels of depressive symptoms, more illness-related anxiety and less openness compared with healthy controls. These data suggest that TCC is associated with adverse psychological factors that may persist well after the acute episode.

## Introduction

Takotsubo cardiomyopathy (TCC) is increasingly recognised in clinical cardiology [1–4] and is characterised by severe left ventricular dysfunction, typically with apical ballooning, combined with symptoms mimicking myocardial infarction [5]. This transient condition usually presents with symptoms including chest pain and/or dyspnoea with ST-segment elevation or T‑wave inversion, and the absence of obstructive coronary artery disease [1]. Unique to this syndrome is the high prevalence of emotional triggers preceding the onset of event [1, 2, 6, 7] with estimates ranging from 26–47 %, compared with physical precipitants in 8–50 % [8–13]. Among the psychological triggers are news of an unexpected death, experiencing fear, and other events that result in intense emotional experiences [2, 14]. TCC was first introduced in the Japanese scientific literature by Sato, Dote and their colleagues in 1990 and the first English-language publications date back to 2000. Multiple terms (over 70) have been used to describe this syndrome, including the typical left ventricular abnormality (e.g., apical ballooning syndrome) and the characteristic psychological precipitants (e.g., stress cardiomyopathy), but it has been argued that TCC is the most appropriate name for this condition [15].

The high prevalence of emotional triggers in TCC suggests that some individuals are particularly vulnerable to experiencing psychological distress that may result in this syndrome. The prevalence of depression and anxiety disorders in TCC ranges from 21–60 % [1, 16–19]. An increase in levels of psychological distress from admission to one-year follow-up is common [20]. Few studies have obtained a comprehensive assessment of psychological measures of distress in patients with TCC.

Illness-related anxiety and related constructs such as hypochondriasis are of particular relevance to TCC because of the unpredictable nature of this syndrome. No studies have evaluated illness-related anxiety or hypochondriasis in TCC. In addition, these distress-related measures have not been studied in the context of patients’ personality factors. Stable personality factors may contribute to individual differences in emotional well-being, responses to distressing factors and cardiovascular reactivity to emotional stress [14].

This study tested whether patients with a clinical history of TCC display higher levels of depressive symptoms, perceived stress and general anxiety and illness-related anxiety compared with healthy controls and patients with chronic heart failure (HF). In addition, we examined whether personality factors play a role in the hypothesised elevated levels of depression, psychological distress and anxiety in TCC patients versus controls. Investigating the psychological correlates of TCC could be useful in optimising patient risk stratification and elucidating the pathophysiological processes involved [14].

## Methods

### Study sample

Between January 2012 and April 2014, 56 patients (18 TCC patients, 19 HF patients and 19 healthy controls) participated in the study. A previous report based on this study addressed the neurohormonal and haemodynamic responses in TCC [22]. The diagnosis was based on 1) akinesia or dyskinesia of the apical and/or mid-ventricular segments of the left ventricle with regional wall motion abnormalities that extend beyond the distribution of a single epicardial vessel; 2) signs and symptoms suggesting acute coronary syndrome (i.e., new-onset ECG abnormalities such as ST-segment elevation and/or T‑wave inversion, modest elevation in cardiac troponin levels, and/or typical angina symptoms); and 3) absence of obstructive coronary artery disease, pheochromocytoma or myocarditis that could account for the condition [1]. TCC patients were identified by review of the electronic medical records over the past 5 years (between 2009 and 2014). Patients were not included if TCC occurred in response to surgery or acute injury. The mean time between the acute event and study participation was 23 ± 18 months (median = 23, IQR = 3–40 months).

A control group of patients with stable HF (NYHA class I–II) without a history of TCC was recruited from the same hospital. This HF control group was used to examine the role of compromise in left ventricular function combined with minimal or no symptoms compared with the normal left ventricular function in TCC. A second control group consisted of healthy women and men who were recruited by advertisement. The healthy controls were required not to have a history of TCC, HF or coronary artery disease.

Exclusion criteria for all participants were: 1) age >85 years, 2) current active treatment for cancer or another life-threatening condition, 3) currently on hormone replacement therapy, and 4) cognitive impairment interfering with completion of questionnaires. The study was conducted at the Elisabeth-TweeSteden Hospital, Tilburg, the Netherlands. The protocol was approved by the Institutional Review Board (#NL35988.008.11). All participants provided informed consent prior to participating.

### Measurements

Depression: Depressive symptoms were assessed using the Patient Health Questionnaire (PHQ‑9) [23]. The items assess the standard criteria for major depressive disorder, with higher scores indicating higher levels of depression. A cut-off score of ≥10 indicates a moderate to severe level of depression symptoms. The internal consistency of the PHQ‑9 is excellent (Cronbach’s α = 0.89).

Perceived stress: The Perceived Stress Scale (PSS) is designed to measure the degree to which situations in one’s life are appraised as stressful [24]. The 10-item version shows better psychometric characteristics in comparison with the original 14-item scale [26] with good reliability characteristics (Cronbach’s α = 0.85) [25].

General anxiety: The 7‑item General Anxiety Disorder (GAD-7) scale was used to measure anxiety symptoms [26]. The GAD-7 has excellent internal consistency (Cronbach’s α = 0.92).

Illness-related anxiety: Illness-related anxiety was assessed using the Whiteley-7 scale. This questionnaire assesses illness-related worries and beliefs and is valid for the evaluation of illness-related anxiety and hypochondriasis in medical settings. The internal consistency of the WI-7 is moderate (Cronbach’s α = 0.68) [27].

Personality factors: Personality factors were assessed using the NEO Five-Factor Inventory (NEO-FFI) [28] and the DS-14 for type-D personality [29]. The NEO-FFI [28] assesses the ‘Big-5’ personality dimensions: neuroticism, extraversion, openness, conscientiousness and agreeableness. The reliability of the NEO-FFI is acceptable to good (Cronbach’s α values ranging from 0.69 to 0.86).

The DS14 assesses the distressed personality type (Type D) and consists of two 7‑item subscales: negative affectivity and social inhibition. The DS14 has a high internal consistency (Cronbach’s α = 0.88 and 0.86 for the two subscales, respectively) [29]. Continuous scores were used to assess the negative affect and social inhibition dimensions. The interaction between the negative affectivity and social inhibition subscales, based on the product of the subscale z‑scores, was used as the primary measure of type D. In addition, the previously validated cut-off score ≥ 10 on both subscales was used to classify the presence or absence of type D personality to enable comparisons with other studies [21, 29].

### Statistical analyses

Data are presented as mean ± standard deviation or N and percentages as appropriate. The square root of the raw values for the PHQ‑9, GAD-7 and WI-7 was used to limit bias related to non-normal distribution of the data prior to statistical analyses. The PHQ‑9 was also dichotomised using the clinical cut-off value ≥ 10 [23].

Differences between the TCC patients and both control groups were examined using independent samples t‑tests and χ^2^ tests. Multivariate analysis of variance (MANOVA) was used to examine differences between TCC and the control groups on the combined set of psychological dependent variables (using general linear models). The final MANOVA models involved analyses adjusted for age and sex and examined whether group differences in depressive symptoms, perceived stress and anxiety remained significant when adjusting for stable personality factors. Data were analysed using SPSS (version 22.0) and a two-sided *p*-value of <0.05 was considered statistically significant.

## Results

Characteristics of the study sample are shown in Table 1. TCC patients were on average older compared with the healthy control group (68.3 ± 11.7 vs. 60.0 ± 7.6 years, *p* = 0.014) and less likely to be employed. A history of clinical depression was present in 6 (33 %) TCC patients, compared with 4 (21 %) in the healthy control group and 6 (32 %) in the HF group (*p* = 0.837). Consistent with the inclusion criteria, left ventricular ejection fraction was normal in TCC (63.6 ± 7.5 %) and healthy controls (64.5 ± 7.5 %) and reduced in the HF control group (38.3 ± 15.4 %). Patients with TCC were more likely to have hypertension and use medications (beta-adrenergic blocking agents, ACE inhibitors, antiplatelet medications, and lipid-lowering drugs) compared with healthy controls (Table 1).

**Table 1:** Patient characteristics

	Takotsubo *N* = 18	Heart failure *N* = 19	TTC vs. HF *p-*value	Healthy control *N* = 19	TTC vs. healthy *p-*value
Female	14 (77.8 %)	13 (68.4 %)	0.522	13 (68.4 %)	0.522
Age	68.3±11.7	68.8±10.1	0.888	60.0±7.6	**0.014**
Married	11 (61.1 %)	14 (77.8 %)	0.278	14 (73.7 %)	0.414
Children	15 (83.3 %)	17 (94.4 %)	0.289	15 (78.9 %)	0.734
Higher education	3 (16.7 %)	2 (11.8 %)	0.679	8 (42.1 %)	0.091
Employed	3 (16.7 %)	3 (16.7 %)	>0.999	13 (68.4 %)	**0.001**
Smoking status	3 (16.7 %)	5 (27.8 %)	0.423	0 (0.0 %)	0.063
Alcohol use	11 (61.1 %)	8 (44.4 %)	0.317	16 (84.2 %)	0.114
BMI (kg/m^2^)	26.19±5.1	29.64±5.2	0.053	26.00±3.9	0.898
**Medical history**					
Atrial fibrillation	1 (5.6 %)	3 (15.8 %)	0.316	0 (0.0 %)	0.298
Hypertension	15 (83.3 %)	17 (89.5 %)	0.585	6 (31.6 %)	**0.001**
Hypercholesterolaemia	8 (44.4 %)	12 (63.2 %)	0.254	3 (15.8 %)	0.057
Diabetes	1 (5.6 %)	9 (47.4 %)	**0.004**	0 (0.0 %)	0.298
Stroke	1 (5.6 %)	1 (5.6 %)	>0.999	0 (0.0 %)	0.298
COPD	1 (5.6 %)	4 (22.2 %)	0.148	0 (0.0 %)	0.298
Cancer	4 (22.2 %)	4 (25.0 %)	0.849	2 (10.5 %)	0.335
Kidney or liver disease	2 (11.1 %)	1 (5.9 %)	0.581	0 (0.0 %)	0.135
Stomach ulcer	2 (11.1 %)	0 (0 %)	0.146	0 (0.0 %)	0.135
**Medication**					
Beta-blockers	12 (75.0 %)	14 (87.5 %)	0.365	3 (15.8 %)	**<0.001**
ACE-inhibitors	11 (61.1 %)	15 (78.9 %)	0.235	1 (5.3 %)	**<0.001**
Diuretics	4 (22.2 %)	16 (84.2 %)	**<0.001**	3 (15.8 %)	0.618
Vasodilators	1 (6.3 %)	8 (50.0 %)	**0.006**	0 (0.0 %)	0.269
Antiplatelet	13 (76.5 %)	16 (100 %)	**0.038**	1 (5.3 %)	**<0.001**
Lipid lowering	7 (41.2 %)	10 (62.5 %)	0.221	2 (10.5 %)	**0.034**
Antidepressants	1 (6.3 %)	2 (12.5 %)	0.544	0 (0.0 %)	0.269
Benzodiazepine	2 (12.5 %)	2 (12.5 %)	>0.999	0 (0.0 %)	0.112

*TCC* takotsubo cardiomyopathy, *HF* heart failure, *BMI* body mass index, *COPD* chronic obstructive pulmonary disease.

A triggering event related to the onset of TCC was reported in 10/18 (56 %) patients with TCC: 8 patients had an emotional trigger, and 2 patients reported a physical trigger combined with psychological distress, 1 patient could not remember the precipitating circumstances, and 7 patients had no clear triggering pre-TCC event.

### Depression, perceived stress and general anxiety as related to TCC

**Fig. 1:**
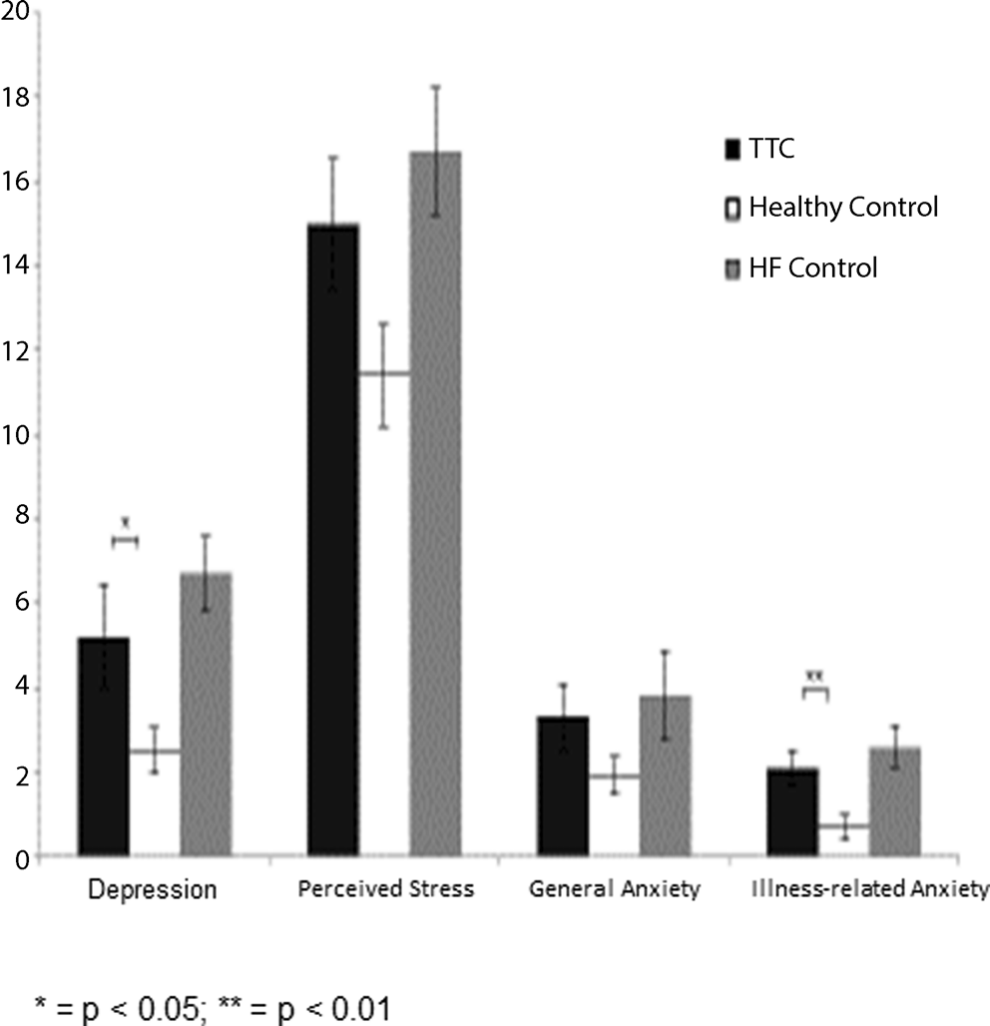
Differences in depressive symptoms, perceived stress, general anxiety and illness-related anxiety between TTC patients, HF patients and healthy controls

As shown in Fig. 1, TTC patients reported higher levels of depressive symptoms compared with healthy controls (5.2 ± 5.2 vs. 2.5 ± 2.4, t(35) = 2.144, *p* = 0.039). There were no differences between TTC patients and HF patients regarding the severity of depressive symptoms (HF: 6.7 ± 3.9, t(35) = −1.501, *p* = 0.142). Moderate to severe depression (PHQ‑9 ≥ 10) was reported by 3 (16.7 %) TTC patients, 0 of the healthy controls and 4 (21.1 %) HF patients (*p* = 0.118).

Perceived stress levels were higher in TTC compared with healthy controls (15.0 ± 6.6 vs. 11.4 ± 5.1) although this difference did not meet the cut-off criteria for statistical significance (t(34) = 1.854, *p* = 0.072). No differences were observed between TTC patients and HF patients (HF: 16.7 ± 6.6, t(34) = −0.704, *p* = 0.486).

General anxiety levels were not markedly higher in TTC patients compared with healthy controls (3.3 ± 3.3 vs. 1.9 ± 1.9, t(35) = 1.401, *p* = 0.170) or HF (HF: 3.8 ± 4.6, t(35) = 0.076, *p* = 0.940).

### *TTC and illness-related anxiety*

Illness-related anxiety levels were higher in TTC patients compared with healthy controls (2.1 ± 1.7 vs. 0.7 ± 1.3, t(32) = 3.037, *p* = 0.005) (Fig. 1). Analyses per item indicated that this association was mainly carried by health anxiety-related concerns: i.e., ‘Do you worry a lot about your health’ (*p* = 0.002) and ‘Do you feel that you are bothered by many different symptoms’ (*p* = 0.004). There were no differences between TTC patients and HF patients regarding illness-related anxiety (HF: 2.6 ± 2.1, t(33) = −0.588, *p* = 0.561).

### Personality factors in TTC versus controls

As shown in Table 2, patients with TTC had significantly lower levels of openness compared with healthy controls (34.2 ± 4.3 vs. 38.2 ± 5.6, t(34) = −2.416, *p* = 0.021). None of the other NEO-based personality traits were significantly different between TTC patients and healthy controls and no differences were observed between TTC patients and HF patients (Table 2).

**Table 2:** Personality factors in takotsubo cardiomyopathy versus heart failure and healthy controls

	Takotsubo *N* = 17	Healthy controls *N* = 19	TTC vs. healthy *p-*value	Heart failure *N* = 19	TTC vs. HF *p-*value
**Big-5**					
Neuroticism	31.0±8.1	29.1±6.8	0.275	31.5±8.5	0.883
Extraversion	40.0±7.1	40.0±6.4	0.985	38.8±6.6	0.583
Openness	34.2±4.3	38.2±5.6	**0.021**	32.7±4.6	0.343
Conscientiousness	46.3±5.1	46.0±5.5	0.972	44.3±5.7	0.290
Agreeableness	45.4±4.3	44.4±4.3	0.513	43.5±5.4	0.258
**Type D**					
Negative affectivity	7.1±6.2	5.5±4.8	0.379	9.4±8.6	0.377
Social inhibition	8.2±7.0	7.9±7.5	0.878	7.5±6.6	0.737
Interaction NA x SI	0.36±1.2	0.34±0.6	0.937	0.40±1.5	0.936

*TCC* takotsubo cardiomyopathy, *HF* heart failure, *NA* negative affectivity, *SI* Social Inhibition, Interaction, based on z‑scores

Regarding type D personality, the subscales negative affectivity and social inhibition did not differ between TTC patients and the control groups and no differences between groups were observed in the model including negative affectivity (*p* = 0.210), social inhibition (*p* = 0.948) and their interaction (*p* = 0.986). Dichotomised type D personality criteria were met by 2 (11.8 %) TTC patients, 3 (15.8 %) healthy controls and 3 (15.8 %) HF patients (*p* = 0.926).

### Multivariate analyses

MANOVA including measures of depressive symptoms, perceived stress, general anxiety and illness-related anxiety revealed a significant difference among groups (Wilks’ Lambda = 0.656, F(8,92) = 2.699, *p* = 0.010, eta^2^ = 0.190) and this remained significant after adjusting for age and sex (*p* = 0.018). MANOVA-based group comparisons for the personality factors were consistent with the aforementioned bivariate analyses, revealing no significant main effect for patient group (Wilks’ Lambda = 0.739, F(12,94) = 1.276, *p* = 0.246, eta^2^ = 0.140; age- and sex-adjusted *p* = 0.532, eta^2^ = 0.109).

The psychological measures were significantly interrelated as shown in Table 3. MANOVA revealed that TTC patients continued to have elevated illness-related anxiety levels compared with healthy controls when adjusting for personality factors, age and sex (*p* = 0.031), whereas the association between TTC and depression was not significant in the personality factor-adjusted model (*p* = 0.432).

**Table 3:** Associations between psychological measures

	1	2	3	4	5	6	7	8	9	10
1. PHQ‑9	1.0									
2. GAD-7	0.598***									
3. PSS-10	0.628**	0.636***								
4. WI-7	0.491***	0.398**	0.468***							
5. Neuroticism	0.587***	0.594***	0.737***	0.334*						
6. Extraversion	−0.325*	−0.221	−0.337*	−0.194	−0.409**					
7. Openness	−0.433**	−0.273*	−0.465***	−0.323*	−0.318*	0.117				
8. Conscientiousness	−0.390***	−0.147	−0.431**	−0.097	−0.357**	0.363**	0.020			
9. Agreeableness	−0.166	−0.080	−0.365**	−0.338*	−0.325*	0.326*	0.057	0.396*		
10. Negative affectivity	0.616***	0.591***	0.633***	0.376**	0.693***	−0.488**	−0.263	−0.318*	−0.330*	
11. Social inhibition	0.259	0.163	0.248	0.122	0.304*	−0.737***	−0.149	−0.239	−0.126	0.367**

*PHQ* Patient Health Questionnaire, *GAD-7* the 7‑item General Anxiety Disorder scale, *PSS-10* 10-item Perceived Stress Scale, *WI-7* Whiteley-7 scale

***p < 0.001; **p < 0.01; *p < 0.05

## Discussion

Patients with TTC displayed higher levels of depressive symptoms and illness-related anxiety compared with healthy controls. General anxiety and perceived stress were not significantly higher in TTC patients. TTC patients also displayed less openness compared with healthy controls, but no other differences were found regarding personality factors. TTC patients did not differ from HF patients on any of the psychological measures. These findings suggest the possibility that the unpredictable nature of TTC and the lack of a sustained underlying cardiac disease process may result in increased illness-related anxiety and depressive symptoms in TTC patients.

The finding that patients with TTC have more illness-related anxiety compared with healthy controls is novel and consistent with observations in other atypical cardiac conditions. Illness-related anxiety and hypochondriasis predict persistent symptoms in unexplained chest pain [30]. Prior studies have reported that TTC [19] and other ‘atypical’ cardiac conditions (non-cardiac chest pain) [31] are associated with general anxiety and/or anxiety disorders. The present investigation showed elevated illness-related anxiety whereas general anxiety was not substantially elevated in TTC. Item analysis of the Whiteley Index indicated that the differences between TTC patients versus controls were mainly carried by illness-related concerns and not an inability to be reassured by physician diagnosis. Increases in levels of psychological distress from admission to one-year follow-up have been found in TTC patients compared with patients with myocardial infarction, despite the improvements in left ventricular function in TTC [20]. The dissociation between echocardiographically determined left ventricular function and symptoms has also been observed in a large sample of general practice patients [32]. These findings may indicate that TTC is followed by increased health concerns over time that may require intervention in selected patients.

Consistent with previous studies, we observed elevated depression levels in TTC. The prevalence of depression disorder prior to TTC ranged between 21 and 36 % in prior studies [18] and was 33 % in the present group of TTC patients. In the present study, levels of depressive symptoms in the post-TTC period were comparable with those reported by the HF comparison group. These findings indicate that TTC is associated with psychological burden well after the acute TTC episode has resolved. It is possible that the integration between the central nervous system and the autonomic nervous system is impaired in TTC, including dysregulation of the central autonomic network such as the medial prefrontal cortex and the insula [33]. This dysregulation may potentially explain the observed elevated mental stress-induced catecholamine response in TTC combined with a blunted emotional arousal response [22]. Post-TTC depressive symptoms and the bio-behavioural correlates of TTC may also interfere with employability, as patients with TTC were less often employed compared with healthy controls.

Of the personality factors examined in this study, we found that TTC patients reported lower levels of openness compared with healthy individuals, but no other differences were found regarding personality factors. The low level of openness in TTC fits our prior observation in this study of blunted emotional arousal responses to mental stress [22]. In contrast to prior observations, no associations between TTC and type D personality were found, which may in part reflect the very high prevalence of type D in the study by Compare et al. (76 % of TTC patients with and 43 % of TTC patients without an emotional trigger) [21] compared with 11.8 % in the present sample. The role of emotional triggers that precede TTC in the subsequent development or maintenance of distress-related psychological measures requires additional research with larger samples that allow sub-group and covariate-adjusted analyses.

Limitations of this study include the following. The sample size of this study is small which limited the number of covariates that could be used in the multivariate models. The TTC patients also had a less favourable cardiovascular risk profile, particularly hypertension, but also diabetes and smoking status and lower education compared with healthy controls. It is therefore possible that these factors confounded the observed association between TTC and psychological measures. The healthy control group was also younger than the TTC group, but statistical adjustment for age had minimal effect on the results. In addition, the time between the acute TTC event and study participation differed within the TTC patients, which may have resulted in biases, although no significant correlations were found between the time since TTC with any of the psychological measures. Selective survival of TTC patients with relatively low psychological burden may have attenuated the observed differences. There are also several strengths to this investigation including the novelty of the assessment of multiple psychological measures, including illness-related anxiety and personality factors in this unique patient group.

In conclusion, levels of illness-related anxiety and depressive symptoms were elevated in TTC patients compared with healthy controls. These differences were not explained by stable personality factors. Additional research is needed to determine the prognostic value of psychological measures, and optimal treatment modules need to be developed to reduce illness-related anxiety and depressive symptoms in patients with TTC.

### CVOI E‑learning formula!

This is the CVOI e‑learning article. The author has prepared 10 questions which are available through the website of the Cardiovascular Educational Institute (CVOI.). Please follow the instructions below.
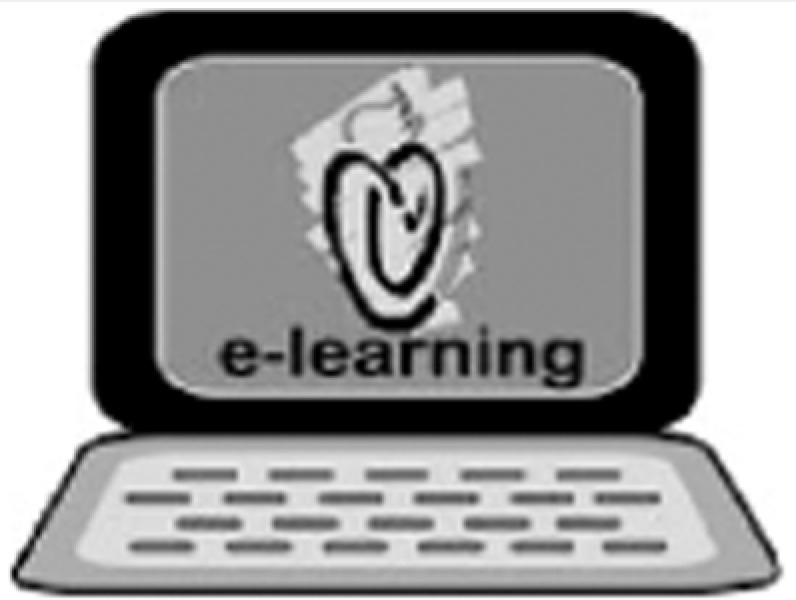


After finishing the questions you will be asked to fill in your name, hospital and e‑mail address; then press the button “verzenden”.

When 6 out of the 10 questions are answered correctly, you acquire 1 accreditation point granted by the Quality Committee of the Netherlands Society of Cardiology (NVVC). The acquired point will be credited to your personal file in the GAIA system. You will also receive an e‑mail with all the correct answers.

Over a period of one year 10 e-leaming articles will appear in 10 subsequent NHJ editions. In each edition the e‑learaing article will be recognisable by a special icon. On an annual basis you can collect 10 accreditation points. The accreditation points are credited in the GAIA system by the CVOI.

If you need additional information, please contact the CVOI by e‑mail: cvoi@cvoi.orgor by phone: 030-2345001.

E.E. van der Wall, Chief editor NHJ

K.B. Schick, Coordinator CVOI
